# Primary care use and cardiovascular disease risk in Russian 40–69 year olds: a cross-sectional study

**DOI:** 10.1136/jech-2019-213549

**Published:** 2020-09-01

**Authors:** Jakob Petersen, Anna Kontsevaya, Martin McKee, Erica Richardson, Sarah Cook, Sofia Malyutina, Alexander V Kudryavtsev, David A Leon

**Affiliations:** 1 London School of Hygiene and Tropical Medicine, London, UK; 2 UCL, London, UK; 3 National Research Centre for Preventive Medicine, Moskva, Russian Federation; 4 Health Services Research and Policy, London School of Hygiene and Tropical Medicine, London, UK; 5 ECOHOST, London School of Hygiene and Tropical Medicine, London, UK; 6 Department of Community Medicine, UiT Arctic University of Norway, Tromso, Norway; 7 Institute of Internal Medicine, Siberian Branch of the Russian Academy of Medical Sciences, Novosibirsk, Russian Federation; 8 Novosibirsk State Medical University, Novosibirsk, Russian Federation; 9 Northern State Medical University, Arkhangelsk, Russian Federation

**Keywords:** Epidemiology, ﻿Public health, Lifestyle, Environmental health, Clinical epidemiology, Cost effective, Disease modelling, Economic evaluation, Eastern Europe, International health, Policy, Health services

## Abstract

**Background:**

The Russian Federation has very high cardiovascular disease (CVD) mortality rates compared with countries of similar economic development. This cross-sectional study compares the characteristics of CVD-free participants with and without recent primary care contact to ascertain their CVD risk and health status.

**Methods:**

A total of 2774 participants aged 40–69 years with no self-reported CVD history were selected from a population-based study conducted in Arkhangelsk and Novosibirsk, Russian Federation, 2015–2018. A range of co-variates related to socio-demographics, health and health behaviours were included. Recent primary care contact was defined as seeing primary care doctor in the past year or having attended a general health check under the 2013 Dispansarisation programme.

**Results:**

The proportion with no recent primary care contact was 32.3% (95% CI 29.7% to 35.0%) in males, 16.3% (95% CI 14.6% to 18.2%) in females, and 23.1% (95% CI 21.6% to 24.7%) overall. In gender-specific age-adjusted analyses, no recent contact was also associated with low education, smoking, very good to excellent self-rated health, no chest pain, CVD 10-year SCORE risk 5+%, absence of hypertension control, absence of hypertension awareness and absence of care-intensive conditions. Among those with no contact: 37% current smokers, 34% with 5+% 10-year CVD risk, 32% untreated hypertension, 20% non-anginal chest pain, 18% problem drinkers, 14% uncontrolled hypertension and 9% Grade 1–2 angina. The proportion without general health check attendance was 54.6%.

**Conclusion:**

Primary care and community interventions would be required to proactively reach sections of 40–69 year olds currently not in contact with primary care services to reduce their CVD risk through diagnosis, treatment, lifestyle recommendations and active follow-up.

## INTRODUCTION

Cardiovascular (CVD) mortality rates, especially among men, have been very high in the Russian Federation.^1 2^ Fortunately, they have been declining since 2005, although they remain considerably higher than in many countries at similar levels of economic development. This decline has been attributed, to a large extent, to success against proximal factors, particularly in hazardous alcohol consumption and smoking, but there is also growing recognition that improvements in healthcare have played a role.

Visits to primary care facilities offer opportunities for early detection of those at risk and for primary prevention, for example, by advising lifestyle modification, treating high blood pressure and using medication to improve lipid profiles. Yet, in all types of health system, there are people who are effectively unknown to the health system, rarely if ever visiting primary care facilities. The reasons are many, but include characteristics of the individuals concerned, including knowledge, health beliefs, proximity and opportunity, as well as those of the health system, such as the presence of geographical, temporal and financial barriers.^3–^ ^5^

The most recent reforms of the healthcare system in the Russian Federation began in 2005, with a renewed emphasis on tackling non-communicable disease and strengthening primary care.^6–8^ The Federal Ministry of Health subscribes to the approach set out in the WHO declarations of Alma-Ata and, more recently, Astana, which advocates a focus on primary care, which has been found to deliver better health outcomes, improved accessibility, cost-containment and reduction of health inequalities in many healthcare systems.^9^ The Russian primary care system is based on polyclinics offering a range of services to patients living in the surrounding communities.^6 7^ However, since 2013, these have been supplemented by a health check programme, *Dispanserizatsiya* (Dispansarisation). This is reminiscent of programmes adopted since the 1920s during the Soviet period.^7^ Every 3 years,^7^ or annually after the age of 40 years,^10^ all persons over 18 years registered with a polyclinic are invited to attend for a general health check. Those found to have either a greater than 5% 10-year CVD risk or diagnosed CVD are invited for further check-ups. There is, however, little published evidence on the reach and impact of the programme.

These two elements of the health system, the network of polyclinics and the large-scale screening programme, offer scope to reduce the burden of CVD in individuals at risk, but there are several potential barriers to maximising any health gain. These include affordability of prescription medicines, which must be paid for out of pocket by many people (although there are widespread exemptions, such as for pensioners)^11 12^; a lack of physicians with primary care training, underfunding of the polyclinic infrastructure with long waiting times﻿; and limited capacity for long-term management of newly detected patients.^6^

A first step in developing a strategy to overcome these challenges is to determine who is being left behind. Here we report on individuals who have not been diagnosed with CVD history in two Russian cities, to ascertain who is or is not in contact with the health system and to understand which groups might benefit from primary preventive interventions but are not doing so.

## METHODS

### Study design and population

Know Your Heart (KYH) is a cross-sectional study of CVD structure, function and risk factors in over 4500 men and women aged 35–69 years in two Russian cities undertaken between 2015 and 2018.^13^ The study was conducted in two stages, with a baseline interview in the home followed by a health check in a local clinic. The response rates for the health check component were 67% in Arkhangelsk and 37% in Novosibirsk. A total of 2774 participants were selected based on the following inclusion criteria: aged 40–69 years; attendance at the health check; and no self-reported history of myocardial infarction, heart failure, atrial fibrillation, angina or stroke events (referred to as ‘CVD-free’ hereinafter). Participants aged less than 40 years were excluded as the SCORE tool used to measure 10-year CVD risk score only covers people aged 40 years and above.^14^

### Outcomes

The main outcome, recent primary care contact, was defined as visiting a primary care doctor in the past year (GP, polyclinic cardiologist or polyclinic specialist) *or* attending a general health check since 2013 (Dispansarisation). A sensitivity analysis was undertaken in which the association with CVD risk class was studied varying the outcome definition on two points. First, by restricting the outcome to having seen a primary care doctor in the past year regardless of having attended a general health check or not. Second, by excluding a small proportion (5.5%) of individuals who had not seen a primary care doctor in the past year, but had seen a hospital doctor in the past year. The latter check was included in recognition of the relatively weak gate-keeping role played by primary care doctors in the Russian Federation.

The mean number of primary care visits per population and proportion of participants with no recent primary care contact were estimated, with standardisation by age and sex using the 2013 European Standard Population.^15^

### Covariates

We identified a range of variables that a priori may be determinants of recent primary healthcare contact. These included measures of socioeconomic position (education and financial constraints), social support (single status), health-related behaviours (smoking, alcohol consumption, physical exercise) and symptoms (angina), and self-reported previous diagnosis of significant care-intensive health conditions (chronic bronchitis, cancer, asthma, rheumatoid arthritis, osteoarthritis, migraine). Levels of education were categorised into elementary (secondary education or less), intermediary (all other than elementary and graduate) and graduate level. Three categories of self-perceived financial constraints were defined: households with individuals who perceived themselves constrained in being able to buy food or clothes, those constrained in buying large domestic appliances, and those not constrained in buying any of the above. Self-reported alcohol consumption and alcohol-related behaviours were jointly categorised into ﻿non-drinker in the past year; low-risk drinkers (score <8); high-risk drinkers (score 8+) according to WHO Alcohol Use Disorder Identification Test (AUDIT) .^16 17^ Physical activity was measured using the Total Physical Activity Index.^18^ The Rose angina questionnaire (short form) captured those with no chest pain, non-anginal chest pain, Grade 1 and 2 angina.^19^ Social support was ascertained as single status as opposed to married or co-habitating.^20^

A series of CVD biomarkers was also included in the analysis. Hypertension was defined as systolic pressure of 140+ mmHg and/or a diastolic of 90+ mmHg or regularly taking antihypertensive medications (International WHO Anatomical Therapeutic Chemical (ATC): C02/3/7/8/9).^21^ Diabetes status was ascertained by either self-reported medical history or taking diabetic medicine (ATC A10) or HbA1c 48+ mmol/mol (>6.5%).^22^ Chronic kidney disease status was defined as estimated glomerular filtration rate below 60 ml/min/1.73 m^2^ based on serum creatinine data.^23^ Serum total cholesterol (mmol/L) was measured and combined with data on age, sex, smoking status and systolic blood pressure to calculate 10-year risk of a fatal CVD event according to the SCORE tool equations for high-risk countries and divided into three risk groups: low (<1%), moderate (1–4.9%) and high (5+%)^14^ (personal communication with Dr A. P. Fitzgerald, University College Cork, Ireland, who provided additional information on the equations underlying the European Cardiology Society’s online CVD risk calculator).

### Statistical analysis

Gender-specific multivariate logistic regression models to explain no recent primary care contact were fitted adjusting for age, education and financial constraints using Stata 15.^24^

### Ethics

Ethical approval was obtained from the ethics committees of the London School of Hygiene & Tropical Medicine (approval number 8808), Novosibirsk State Medical University (approval number 75; 21 May 2015), the Institute of Preventative Medicine (approval received 26 December 2014), Novosibirsk and the Northern State Medical University, Arkhangelsk (approval number 01/01-15; 27 January 2015). The study was conducted in accordance with the 1964 Helsinki Declaration and its later amendments. Participants gave written informed consent.

## RESULTS

The characteristics of study participants are reported in Tables S1, S3-S4 (see supplementary materials). The mean age-standardised numbers of primary care visits in the previous year by 40–69 year olds were 2.4 (95% CI 2.2 to 2.5) in males, 3.4 (95% 3.2 to 3.5) in females and 3.0 (95% 2.9 to 3.1) overall (Table S2). The age-standardised proportion of CVD-free 40–69 year olds with no recent primary contact (no visit to primary care doctor nor general health check attendance since 2013) was 32.3% (95% CI 29.7% to 35.0%) in males, 16.3% (95% CI 14.6% to 18.2%) in females and 23.1% (95% CI 21.6% to 24.7%) overall. The percentages of CVD-free 40–69 year olds who had not attended the Dispansarisation health check (regardless of any of other form of primary care use) were 69.1% (66.4–71.6) among males, 44.0% (41.6–46.4) among females and 54.6% (52.8–56.5) overall. Among those with no contact, 37% were current smokers, 33% had a 5+% 10-year CVD risk, 31% untreated hypertension, 20% non-anginal chest pain, 18% were problem drinkers (AUDIT 8+), 14% had uncontrolled hypertension and 9% Grade 1–2 angina (Table S1).

10.1136/jech-2019-213549.supp1 Supplementary data

Factors associated with no recent primary care contact were male gender (Table S2). In gender-specific age-adjusted analyses, other factors associated with no recent contact were low education, smoking, self-rated general health reported as very good to excellent, no chest pain, CVD 10-year SCORE risk 5+%, uncontrolled hypertension, unaware of having hypertension and no care-intensive conditions (tables 1–2).

**Table 1:** Multivariable logistic regression analysis of no recent primary care contact (primary care visit in the past year or recent general health check attendance) vs those with contact in CVD-free 40- to 69- year-old males: adjusted ORs for gender/age and gender/age/education/financial constraints

Co-variates	Level	OR (adjusted for age)	p Value	OR (adjusted for age/education/financial)	p Value
Education level	1. Elementary only 2. Intermediary 3. Graduate	Ref 0.83 (0.59;1.17) **0.69** (0.48;0.98)	0.293 0.041		
Household financial constraints	1. Constrained 2. Intermediary 3. Relatively unconstrained	Ref 0.84 (0.58;1.21) 0.79 (0.54;1.16)	0.356 0.227		
Single	0. No 1. Yes	Ref 0.95 (0.68;1.33)	0.767	Ref 0.94 (0.67;1.33)	0.725
Smoking status	1. Never smoker 2. Ex-smoker 3. Current smoker	Ref 0.90 (0.65;1.24) **1.44** (1.06;1.96)	0.523 0.021	Ref 0.89 (0.64;1.25) **1.39** (1.00;1.91)	0.509 0.047
Alcohol use disorder level	1. Non-drinker past year 2. Low (AUDIT score <8) 3. High (AUDIT score 8+)	Ref 1.33 (0.90;1.96) 1.32 (0.86;2.02)	0.150 0.207	Ref 1.44 (0.96;2.14) 1.39 (0.90;2.17)	0.077 0.139
Alcohol intake	1. Non-drinker 2. <2.5 g/day 3. 2.5–6.9 g/day 4. 7–14.9 g/day 5. 15–20.9 g/day 6. 21+g/day	Ref 1.41 (0.89;2.23) 1.51 (0.99;2.32) 1.32 (0.83;2.09) 0.99 (0.56;1.75) 1.16 (0.74;1.84)	0.143 0.058 0.238 0.967 0.516	Ref 1.54 (0.95;2.49) **1.61** (1.04;2.49) 1.41 (0.89;2.27) 1.08 (0.60;1.93) 1.21 (0.76;1.94)	0.077 0.033 0.146 0.800 0.424
Physical activity	0. Inactive 1. Moderately inactive 2. Moderately active 3. Active	Ref 0.82 (0.43;1.57) 0.96 (0.55;1.65) 0.83 (0.47;1.46)	0.547 0.871 0.520	Ref 0.81 (0.42;1.56) 0.97 (0.55;1.70) 0.78 (0.43;1.39)	0.523 0.920 0.392
BMI category	1. Normal/underweight (<25 kg/m^2^) 2. Overweight (25–29 kg/m2) 3. Obese (30+ kg/m^2^)	Ref 0.84 (0.63;1.12) 1.09 (0.78;1.50)	0.244 0.618	Ref 0.89 (0.66;1.20) 1.12 (0.80;1.56)	0.443 0.515
Self-rated general health Very good-Excellent	0. Poor/Fair/Good 1. Very good/Excellent	Ref **1.80** (1.39;2.33)	<0.001	Ref **1.84** (1.41;2.39)	<0.001
Chest pain	1. No chest pain 2. Non-anginal chest pain 3. Grade 1 angina 4. Grade 2 angina	Ref **0.69** (0.50;0.93) 0.72 (0.40;1.30) 0.52 (0.11;2.53)	0.016 0.275 0.420	Ref **0.69** (0.51;0.94) 0.74 (0.41;1.34) 0.50 (0.10;2.44)	0.020 0.321 0.392
CVD risk score	1. <1% 2. 1–4.9% 3. 5+%	Ref 1.03 (0.67;1.59) **2.08** (1.15;3.76)	0.890 0.016	Ref 0.98 (0.63;1.53) **2.05** (1.11;3.76)	0.945 0.021
Hypertension	1. Normotensive 2. Controlled hypertension 3. Uncontrolled hypertension 4. Untreated hypertension	Ref **0.38** (0.22;0.64) 0.71 (0.48;1.03) 1.22 (0.91;1.62)	<0.001 0.073 0.182	Ref **0.38** (0.23;0.66) 0.70 (0.48;1.03) 1.19 (0.89;1.60)	0.001 0.074 0.238
Hypertension awareness	0. No 1. Yes	Ref **0.59** (0.46;0.76)	<0.001	Ref **0.60** (0.46;0.78)	<0.001
Diabetic*	0. Not diabetic 1. Diabetic	Ref 0.61 (0.35;1.06)	0.077	Ref 0.63 (0.36;1.10)	0.105
Chronic kidney disease status	0. Normal 1. Reduced filtration rate	Ref 0.79 (0.33;1.90)	0.602	Ref 0.71 (0.28;1.79)	0.463
Care-intensive diseases†	0. No 1. Yes	Ref **0.72** (0.55;0.94)	0.017	Ref **0.71** (0.54;0.93)	0.014

*Diabetic defined as self-reported disease, medication, or elevated HbA1c.

†Care-intensive conditions included CKD, chronic bronchitis, cancer, asthma, rheumatoid arthritis, osteoarthritis, migraine, opioid analgesics use.

AUDIT, Alcohol Use Disorder Identification Test; BMI, body mass index; CKD, chronic kidney disease; CVD, cardiovascular disease; HbA1c, hemoglobin A1c.

**Table 2:** Multivariable logistic regression analysis of no recent primary care contact (primary care visit in past year or recent general health check attendance) versus those with contact in CVD-free 40- to 69-year-old females: adjusted ORs for gender/age and gender/age/education/financial constraints

Co-variates	Level	OR (adjusted for age)	p Value	OR (adjusted for age/education/financial)	p Value
Education level	1. Elementary only 2. Intermediary 3. Graduate	Ref **0.60** (0.39;0.91) **0.57** (0.37;0.89)	0.017 0.013		
Household financial constraints	1. Constrained 2. Intermediary 3. Relatively unconstrained	Ref **0.70** (0.50;0.99) 0.76 (0.52;1.11)	0.048 0.157		
Single	0. No 1. Yes	Ref 0.97 (0.74;1.28)	0.835	Ref 0.95 (0.71;1.27)	0.738
Smoking status	1. Never smoker 2. Ex-smoker 3. Current smoker	Ref 0.93 (0.63;1.37) **1.73** (1.24;2.41)	0.721 0.001	Ref 0.92 (0.62;1.36) **1.67** (1.18;2.36)	0.666 0.004
Alcohol use disorder level	1. Non-drinker past year 2. Low (AUDIT score <8) 3. High (AUDIT score 8+)	Ref 1.00 (0.64;1.58) 1.81 (0.79;4.12)	0.988 0.158	Ref 1.00 (0.64;1.60) 1.72 (0.75;3.94)	0.971 0.202
Alcohol intake	1. Non-drinker 2. <2.5 g/day 3. 2.5–6.9 g/day 4. 7–14.9 g/day 5. 15–20.9 g/day 6. 21+g/day	Ref 1.02 (0.64;1.61) 0.95 (0.57;1.60) 1.16 (0.59;2.30) 1.06 (0.37;3.10) 1.79 (0.67;4.78)	0.947 0.852 0.662 0.911 0.245	Ref 1.03 (0.65;1.65) 0.94 (0.55;1.60) 1.14 (0.57;2.30) 1.04 (0.35;3.05) 1.81 (0.67;4.88)	0.890 0.822 0.707 0.946 0.239
Physical activity	0. Inactive 1. Moderately inactive 2. Moderately active 3. Active	Ref 1.44 (0.75;2.75) 1.21 (0.70;2.09) 1.25 (0.70;2.24)	0.277 0.500 0.443	Ref 1.37 (0.70;2.68) 1.14 (0.65;2.02) 1.13 (0.62;2.09)	0.356 0.645 0.689
BMI category	1. Normal/underweight (<25 kg/m^2^) 2. Overweight (25–29 kg/m^2^) 3. Obese (30+ kg/m^2^)	Ref 0.84 (0.60;1.18) 1.04 (0.75;1.44)	0.308 0.837	Ref 0.79 (0.55;1.12) 0.99 (0.71;1.39)	0.184 0.950
Self-rated general health Very good-Excellent	0. Poor/Fair/Good 1. Very good/Excellent	Ref **1.48** (1.13;1.95)	0.005	Ref **1.63** (1.23;2.16)	0.001
Chest pain	1. No chest pain 2. Non-anginal chest pain 3. Grade 1 angina 4. Grade 2 angina	Ref **0.63** (0.45;0.88) 0.85 (0.56;1.28) **0.22** (0.05;0.94)	0.006 0.442 0.041	Ref **0.64** (0.45;0.90) 0.87 (0.57;1.32) **0.23** (0.05;0.95)	0.009 0.503 0.043
CVD risk score	1. <1% 2. 1–4.9% 3. 5+%	Ref 1.05 (0.65;1.69) **2.58** (1.27;5.23)	0.852 0.009	Ref 1.08 (0.66;1.76) **2.49** (1.20;5.16)	0.766 0.014
Hypertension	1. Normotensive 2. Controlled hypertension 3. Uncontrolled hypertension 4. Untreated hypertension	Ref **0.39** (0.24;0.62) 0.87 (0.57;1.31) **1.51** (1.04;2.19)	<0.001 0.503 0.029	Ref **0.38** (0.24;0.62) 0.80 (0.52;1.24) **1.49** (1.02;2.19)	<0.001 0.320 0.039
Hypertension awareness	0. No 1. Yes	Ref **0.58** (0.43;0.79)	<0.001	Ref **0.54** (0.40;0.74)	<0.001
Diabetic*	0. Not diabetic 1. Diabetic	Ref 0.74 (0.44;1.27)	0.248	Ref 0.75 (0.45;1.27)	0.292
Chronic kidney disease status	0. Normal 1. Reduced filtration rate	Ref **1.84** (1.00;3.39)	0.049	Ref 1.77 (0.94;3.32)	0.075
Care-intensive diseases†	0. No 1. Yes	Ref **0.57** (0.43;0.76)	<0.001	Ref **0.57** (0.43;0.76)	<0.001

*Diabetic defined as self-reported disease, medication, or elevated HbA1c.

†Care-intensive conditions included CKD, chronic bronchitis, cancer, asthma, rheumatoid arthritis, osteoarthritis, migraine, opioid analgesics use.

AUDIT, Alcohol Use Disorder Identification Test; BMI, body mass index; CKD, chronic kidney disease; CVD, cardiovascular disease; HbA1c, hemoglobin A1c.

The results of the sensitivity analyses were consistent with the main analyses (Table S5).

## DISCUSSION

A high proportion of CVD-free 40–69 year olds in the current study had no recent primary care contact, that is, 32% of males and 16% of females. Among them, 46% of males and 13% of females had a 10-year CVD risk score at or in excess of 5%. This reflected high levels of untreated hypertension (39% of males and 21% of females) and uncontrolled hypertension (14% of males and 15% of females). These findings are concerning as hypertension is a major risk factor for premature death and CVD morbidity.^21 25 26^ As it is an asymptomatic condition, it is unlikely to be detected, let alone treated or controlled unless the individual concerned comes into contact with the health system.

Factors associated with no recent primary care contact were male gender, younger ages, low education, smoking, very good to excellent self-rated health, no chest pain, CVD 10-year SCORE risk 5+%, absence of hypertension control, absence of hypertension awareness and absence of care-intensive conditions. These findings are largely consistent with the existing literature on factors associated with primary care consultation (age, gender^27^ and smoking^28^) and participation in general health checks.^4^

Although the Dispansarisation scheme is seen as an important element of the Russian prevention strategy, 69% of males and 44% of females had not attended. Some caution is needed in interpreting these findings as the 2013 programme had yet to be fully implemented in the two cities studied when data collection began in 2015. Under the new legislation, enacted in 2019, both primary care and the Dispansarisation programme are being strengthened.^10 29^ Dispansarisation is now open to those aged 40 years and above for annual appointments and three-yearly for younger age groups. The new federal, ‘Primary Care’ programme, now being rolled out, enables users to make appointments over the Internet, extends opening hours to evenings and Saturdays and seeks to improve access for rural populations.^29^

Our study found that 40- to 69﻿-year﻿-old males made, on average, 2.4 (95% CI 2.2 to 2.5) visits per year, with 3.4 (95% CI 3.2 to 3.5) by females and 3.0 (95% CI 2.9 to 3.1) overall. These estimates were very close (2.3 in males and 2.6 in females) to those reported by the concurrent Epidemiology of Cardiovascular Diseases and Its Risk Factors in Some Regions of the Russian Federation (ESSE-RF), a large, cross-sectional, multicentre, population-based study of 25–64 year olds.^30^ The WHO Health for All programme, in contrast, estimated outpatient contacts per person per year in 2015 to be 9.5.^31^ There could be several reasons why we obtained lower estimates than WHO, including study population selection parameters. Consultation rates are generally higher in young children, women of reproductive age and older people^27^; all groups that are entirely or predominantly excluded from the present study; although ESSE-RF did include women of reproductive age. Similarly, KYH is likely to have excluded individuals too ill to leave their home for the health assessment in a local clinic or even to be interviewed. It should be noted that WHO counts all contacts, including home visits, thus inflating the figures compared to KYH and ESSE-RF. Finally, KYH, by design, excludes institutionalised individuals, irregular migrants and individuals with no fixed abode.

Our study has several potential limitations. We used CVD 10-year estimates based on the SCORE tool for high-risk countries, which was accessible to health professionals at the time of the data collection. It should be noted that a newer tool calibrated with data on Eastern European populations is now available.^32^

Sampling bias introduced by non-response is an ever-present problem in surveys. We compared our data against data from the Census 2010 by age, gender and higher education attainment.^13^ Overall, there was very little difference, with a ratio of 0.99 (95% CI 0.93 to 1.06) for Arkhangelsk and 1.26 (95% CI 1.17 to 1.34) for Novosibirsk. In Arkhangelsk, younger participants were however more likely to be educated to degree level than the Census population and older participants less so. Those with more education are generally more likely to be aware of the benefits of preventive measures and the fact that more people with higher education took part in the study could therefore have led to an under-estimation of the public health burden. This must be borne in mind when interpreting the results.

The variables assessed as outcomes and covariates relied on self-reported information and, as such, are subject to reporting biases such as recall and social desirability. Information used in this study was generally not of sensitive nature and concerned the participant’s current or recent situation. As a strength, the 10-year CVD risk estimates were based on objectively measured parameters such as blood pressure and blood cholesterol.

Participants in health surveys are known, in the literature, to underreport primary care contacts relative to secondary care contacts^33^ and the prevalence of primary care contact in this and similar studies could thus be underestimated for that reason alone.

## CONCLUSION

Primary care and community interventions should reach everyone. However, a proportion of CVD-free 40–69 year olds in the Russian Federation are not in contact with primary care services where they can obtain advice and treatment to reduce their CVD risk. A substantial number of them had 5+% 10-year CVD risk (SCORE), untreated hypertension, uncontrolled hypertension and angina amenable to primary preventive interventions. Reforms of primary care services and the Dispansarisation programme for general health checks initiated in 2019 should be closely monitored to ensure that they achieve the improvements in coverage they seek.

**What is already known on this subject**

- Nearly one in four new cardiovascular disease (CVD) cases reported in the European WHO region in 2015 were in the Russian Federation. Hypertension is a major risk factor for CVD and early detection, treatment, and control of hypertension are therefore essential for reducing the burden of CVD. Due to the asymptomatic nature of hypertension, it can only be detected by measuring blood pressure. Primary care is free at the point of use, and local populations are routinely offered general health checks including measurements of blood pressure and other CVD risk factors.

**What this study adds**

- Many 40- to 69-year-old Russians without CVD are currently not in contact with their primary care services. This group had high 10-year CVD risk, and high prevalence of untreated hypertension, uncontrolled hypertension and angina. Primary care and community interventions would be required to proactively reach this group to reduce their CVD risk through diagnosis, treatment, lifestyle recommendations and active follow-up. Current interventions, mainly the general health check programme, should be evaluated carefully to ensure improvements in coverage and equitability for CVD prevention.
