# Supplementary material for: Primary care use and cardiovascular disease risk in Russian 40–69 year olds: a cross-sectional study
Source: J Epidemiol Community Health. 2020 Sep 1;74(9):692–967. doi: 10.1136/jech-2019-213549 (PMC7577087; doi:10.1136/jech-2019-213549)
Supplement: Supplementary data [file jech-2019-213549s001.pdf]

## Supplementary materials - Tables

**Table S1** Characteristics of 40-69 year olds with and without recent primary care contact (primary care visit past year or recent general health check attendance).

| Theme | Co-variate | Contact | | No contact | |
|---|---|---|---|---|---|
| | | N | % | N | % |
| Demographics | Total | 2,134 | 100 | 640 | 100 |
| | Male | 802 | 37.6 | 380 | 59.4 |
| | Female | 1,332 | 62.4 | 260 | 40.6 |
| | | | | | |
| | 40-44yr | 354 | 16.6 | 131 | 20.5 |
| | 45-49yr | 382 | 17.9 | 107 | 16.7 |
| | 50-54yr | 371 | 17.4 | 122 | 19.1 |
| | 55-59yr | 359 | 16.8 | 111 | 17.3 |
| | 60-64yr | 350 | 16.4 | 99 | 15.5 |
| | 65-69yr | 318 | 14.9 | 70 | 10.9 |
| | | | | | |
| | Mean age (SD) | 54.6 | (8.5) | 53.6 | (8.4) |
| | | | | | |
| Health check | Dispansarisation | | | | |
| | No | 873 | 40.9 | 640 | 100 |
| | Yes | 1,261 | 59.1 | 0 | 0 |
| | | | | | |
| Socio-economics | Education | | | | |
| | Elementary only | 246 | 11.5 | 106 | 16.6 |
| | Intermediary | 1,031 | 48.3 | 306 | 47.8 |
| | Graduate | 857 | 40.2 | 228 | 35.6 |
| | | | | | |
| | Economic activity | | | | |
| | Paid work | 767 | 36.0 | 277 | 43.3 |
| | Looking after home | 151 | 7.1 | 33 | 5.2 |
| | Unemployed | 58 | 2.7 | 16 | 2.5 |
| | Retired | 1,116 | 52.3 | 294 | 45.9 |
| | Other | 41 | 1.9 | 20 | 3.1 |
| | | | | | |
| | Financial constraints | | | | |
| | Constrained | 362 | 17.2 | 118 | 19.2 |
| | Intermediary | 1,037 | 49.2 | 286 | 46.4 |
| | Relatively unconstrained | 707 | 33.6 | 212 | 34.4 |
| | | | | | |
| Social support | Single | | | | |
| | No | 1,465 | 68.7 | 481 | 75.2 |
| | Yes | 669 | 31.4 | 159 | 24.8 |
| | | | | | |
| Smoking | Smoking status | | | | |
| | Never smoker | 1,122 | 52.7 | 251 | 39.2 |
| | Ex-smoker | 526 | 24.7 | 154 | 24.1 |
| | Current smoker | 480 | 22.6 | 235 | 36.7 |
| | | | | | |
| Alcohol | Alcohol use disorder | | | | |
| | Non-drinker (past year) | 252 | 11.9 | 67 | 10.5 |
| | Low (Audit score <8) | 1,640 | 77.1 | 457 | 71.7 |
| | High (Audit score 8+) | 234 | 11.0 | 113 | 17.7 |
| | | | | | |
| | Alcohol intake | | | | |
| | Non-drinker | 252 | 11.8 | 67 | 10.5 |
| | <2.5g/day | 920 | 43.2 | 216 | 33.8 |
| | 2.5-6.9g/day | 482 | 22.6 | 162 | 25.4 |
| | 7-14.9g/day | 210 | 9.6 | 87 | 13.6 |

| | | | | | |
|---|---|---|---|---|---|
| | 15-20.9g/day | 93 | 4.4 | 31 | 4.9 |
| | 21+g/day | 174 | 8.2 | 76 | 11.9 |
| Exercise | Physical activity | | | | |
| | Inactive | 143 | 6.8 | 39 | 6.2 |
| | Moderately inactive | 212 | 10.1 | 67 | 10.7 |
| | Moderately active | 1,168 | 55.4 | 335 | 53.3 |
| | Active | 587 | 27.8 | 187 | 29.8 |
| Health | BMI class | | | | |
| | Normal/under weight | 674 | 31.6 | 213 | 33.4 |
| | Overweight | 811 | 38.0 | 229 | 35.9 |
| | Obese | 648 | 30.4 | 196 | 30.7 |
| | Self-rated general health | | | | |
| | Poor/fair/good | 1,178 | 55.3 | 254 | 39.7 |
| | Very good/excellent | 951 | 44.7 | 386 | 60.3 |
| | Chest pain | | | | |
| | No chest pain | 1,244 | 59.7 | 442 | 71.1 |
| | Non-anginal chest pain | 572 | 27.5 | 127 | 20.4 |
| | Grade 1 angina | 218 | 10.5 | 49 | 7.9 |
| | Grade 2 angina | 49 | 2.4 | 4 | 0.6 |
| | CVD risk 10yr SCORE | | | | |
| | <1% | 743 | 35.4 | 193 | 30.8 |
| | 1-4.9% | 899 | 42.8 | 227 | 36.3 |
| | 5+% | 458 | 21.8 | 206 | 32.9 |
| | Hypertension class | | | | |
| | Normotensive | 931 | 44.1 | 298 | 47.3 |
| | Controlled hypertension | 404 | 19.1 | 43 | 6.8 |
| | Uncontrolled hypertension | 372 | 17.6 | 91 | 14.4 |
| | Untreated hypertension | 404 | 19.1 | 198 | 31.4 |
| | Hypertension aware | | | | |
| | No | 1,139 | 53.4 | 429 | 67.0 |
| | Yes | 995 | 46.6 | 211 | 33.0 |
| | Diabetic | | | | |
| | No | 1,940 | 90.9 | 605 | 94.5 |
| | Yes | 194 | 9.1 | 35 | 5.5 |
| | CKD | | | | |
| | No | 2,062 | 96.6 | 618 | 96.6 |
| | Yes | 72 | 3.4 | 22 | 3.4 |
| | Any care-intensive condition[a] | | | | |
| | No | 1,106 | 51.8 | 427 | 66.7 |
| | Yes | 1,028 | 48.2 | 213 | 33.3 |
| Medication | Polypharmacy 5+rx | | | | |
| | No | 2,015 | 94.4 | 635 | 99.2 |
| | Yes | 119 | 5.6 | 5 | 0.8 |
| | Non-opioid analgesics | | | | |
| | No | 2,105 | 98.6 | 632 | 98.8 |
| | Yes | 29 | 1.4 | 8 | 1.3 |

[a]) Care-intensive conditions included: CKD, chronic bronchitis, cancer, asthma, rheumatoid arthritis, osteoarthritis, migraine, opioid analgesics use.

**Table S2** Mean number of primary care visits past year in 40-69 year olds and proportion% of CVD-free with no recent primary care contact standardised to 2013 European Standard Population and Male-Female odds ratios adjusted for age or age, education, and financial constraints.

| Metric | N | Mean (median) | Proportion % | SE | 95% CI | Male-Female OR (age) | 95% CI | Male-Female OR (age/education/financial constraints) | 95% CI |
|---|---|---|---|---|---|---|---|---|---|
| Mean number of primary care visits past year in 40-69 year olds | | | | | | | | | |
| Male | 1,591 | 2.37 (1) | - | 0.08 | 2.23-2.52 | - | - | - | - |
| Female | 2,191 | 3.36 (2) | - | 0.07 | 3.23-3.50 | - | - | - | - |
| Total | 3,782 | 2.95 (2) | - | 0.05 | 2.85-3.05 | - | - | - | - |
| Proportion of CVD-free 40-69 year olds population with no primary care visit past year nor recent general health check attendance (Dispansarisation)% | | | | | | | | | |
| Male | 1,182 | - | 32.3% | 1.33 | 29.7-35.0 | - | - | - | - |
| Female | 1,592 | - | 16.3% | 0.90 | 14.6-18.2 | - | - | - | - |
| Total | 2,774 | - | 23.1% | 0.78 | 21.6-24.7 | 2.45 | 2.05-2.94 | 2.48 | 2.05-2.98 |
| Proportion of CVD-free 40-69 year olds population with no recent general health check attendance (Dispansarisation)% | | | | | | | | | |
| Male | 1,182 | - | 69.1% | 1.33 | 66.4-71.6 | - | - | - | - |
| Female | 1,592 | - | 44.0% | 1.24 | 41.6-46.4 | - | - | - | - |
| Total | 2,774 | - | 54.6% | 0.94 | 52.8-56.5 | 2.86 | 2.44-3.36 | 2.90 | 2.46-3.41 |

**Table S3** Characteristics of 40-69 year old males with and without recent primary care contact (primary care visit past year or recent general health check attendance).

| Theme | Co-variate | Contact | | No contact | |
|---|---|---|---|---|---|
| | | N | % | N | % |
| Demographics | Total | 802 | 100 | 380 | 100 |
| | 40-44yr | 117 | 14.6 | 73 | 19.2 |
| | 45-49yr | 148 | 18.5 | 60 | 15.8 |
| | 50-54yr | 138 | 17.2 | 73 | 19.2 |
| | 55-59yr | 134 | 16.7 | 66 | 17.4 |
| | 60-64yr | 139 | 17.3 | 66 | 17.4 |
| | 65-69yr | 126 | 15.7 | 42 | 11.1 |
| | Mean age (SD) | 54.9 | (8.5) | 54.0 | (8.4) |
| Health check | Dispansarisation | | | | |
| | No | 432 | 53.9 | 380 | 100 |
| | Yes | 370 | 46.1 | 0 | 0.0 |
| Socio-economics | Education | | | | |
| | Elementary only | 125 | 15.6 | 71 | 18.7 |
| | Intermediary | 371 | 46.3 | 184 | 48.4 |
| | Graduate | 306 | 38.2 | 125 | 32.9 |
| | Economic activity | | | | |
| | Paid work | 323 | 40.3 | 170 | 44.8 |
| | Looking after home | 17 | 2.1 | 10 | 2.6 |
| | Unemployed | 36 | 4.5 | 13 | 3.4 |
| | Retired | 405 | 50.5 | 171 | 45.0 |
| | Other | 21 | 2.6 | 16 | 4.2 |
| | Financial constraints | | | | |
| | Constrained | 109 | 13.9 | 59 | 16.1 |
| | Intermediary | 369 | 47.0 | 172 | 46.9 |
| | Relatively unconstrained | 307 | 39.1 | 136 | 37.1 |
| Social support | Single | | | | |
| | No | 675 | 84.2 | 322 | 84.7 |
| | Yes | 127 | 15.8 | 58 | 15.3 |
| Smoking | Smoking status | | | | |
| | Never smoker | 219 | 27.4 | 94 | 24.7 |
| | Ex-smoker | 304 | 38.1 | 116 | 30.5 |
| | Current smoker | 276 | 34.5 | 170 | 44.7 |
| Alcohol | Alcohol use disorder | | | | |
| | Non-drinker (past year) | 116 | 14.5 | 42 | 11.1 |
| | Low (Audit score <8) | 477 | 59.8 | 233 | 61.8 |
| | High (Audit score 8+) | 205 | 25.7 | 102 | 27.1 |

| | | | | | |
|---|---|---|---|---|---|
| | Alcohol intake | | | | |
| | Non-drinker | 116 | 14.5 | 42 | 11.1 |
| | <2.5g/day | 132 | 16.5 | 67 | 17.7 |
| | 2.5-6.9g/day | 190 | 23.7 | 106 | 28.0 |
| | 7-14.9g/day | 137 | 17.1 | 69 | 18.2 |
| | 15-20.9g/day | 71 | 8.9 | 26 | 6.9 |
| | 21+g/day | 156 | 19.5 | 69 | 18.2 |
| | | | | | |
| Exercise | Physical activity | | | | |
| | Inactive | 41 | 5.2 | 22 | 5.9 |
| | Moderately inactive | 87 | 10.9 | 37 | 9.9 |
| | Moderately active | 397 | 49.9 | 193 | 51.7 |
| | Active | 271 | 34.1 | 121 | 32.4 |
| | | | | | |
| Health | BMI class | | | | |
| | Normal/under weight | 238 | 29.7 | 119 | 31.4 |
| | Overweight | 371 | 46.3 | 156 | 41.2 |
| | Obese | 193 | 24.1 | 104 | 27.4 |
| | | | | | |
| | Self-rated general health | | | | |
| | Poor/fair/good | 386 | 48.3 | 128 | 33.7 |
| | Very good/excellent | 414 | 51.8 | 252 | 66.3 |
| | | | | | |
| | Chest pain | | | | |
| | No chest pain | 540 | 68.5 | 283 | 75.7 |
| | Non-anginal chest pain | 198 | 25.1 | 73 | 19.5 |
| | Grade 1 angina | 43 | 5.5 | 16 | 4.3 |
| | Grade 2 angina | 7 | 0.9 | 2 | 0.5 |
| | | | | | |
| | CVD risk 10yr SCORE | | | | |
| | <1% | 90 | 11.4 | 49 | 13.2 |
| | 1-4.9% | 364 | 46.1 | 150 | 40.4 |
| | 5+% | 336 | 42.5 | 172 | 46.4 |
| | | | | | |
| | Hypertension class | | | | |
| | Normotensive | 303 | 38.3 | 158 | 42.1 |
| | Controlled hypertension | 100 | 12.6 | 19 | 5.1 |
| | Uncontrolled hypertension | 153 | 19.3 | 53 | 14.1 |
| | Untreated hypertension | 235 | 29.7 | 145 | 38.7 |
| | | | | | |
| | Hypertension aware | | | | |
| | No | 431 | 53.7 | 254 | 66.8 |
| | Yes | 371 | 46.3 | 126 | 33.2 |
| | | | | | |
| | Diabetic | | | | |
| | No | 742 | 92.5 | 363 | 95.5 |
| | Yes | 60 | 7.5 | 17 | 4.5 |

| | | | | | |
|---|---|---|---|---|---|
| | CKD | | | | |
| | No | 782 | 97.5 | 373 | 98.2 |
| | Yes | 20 | 2.5 | 7 | 1.8 |
| | | | | | |
| | Any care-intensive condition[a] | | | | |
| | No | 513 | 64.0 | 272 | 71.6 |
| | Yes | 289 | 36.0 | 108 | 28.4 |
| | | | | | |
| Medication | Polypharmacy 5+rx | | | | |
| | No | 767 | 95.6 | 377 | 99.2 |
| | Yes | 35 | 4.4 | 3 | 0.8 |
| | | | | | |
| | Non-opioid analgesics | | | | |
| | No | 790 | 98.5 | 376 | 99.0 |
| | Yes | 12 | 1.5 | 4 | 1.1 |

[a]) Care-intensive conditions included: CKD, chronic bronchitis, cancer, asthma, rheumatoid arthritis, osteoarthritis, migraine, opioid analgesics use.

**Table S4** Characteristics of 40-69 year old females with and without recent primary care contact (primary care visit past year or recent general health check attendance).

| Theme | Co-variate | Contact | | No contact | |
|---|---|---|---|---|---|
| | | N | % | N | % |
| Demographics | Total | 1,332 | 100 | 260 | 100 |
| | 40-44yr | 237 | 17.8 | 58 | 22.3 |
| | 45-49yr | 234 | 17.6 | 47 | 18.1 |
| | 50-54yr | 233 | 17.5 | 49 | 18.9 |
| | 55-59yr | 225 | 16.9 | 45 | 17.3 |
| | 60-64yr | 211 | 15.8 | 33 | 12.7 |
| | 65-69yr | 192 | 14.4 | 28 | 10.8 |
| | Mean age (SD) | 54.4 | (8.5) | 53.0 | (8.4) |
| Health check | Dispansarisation | | | | |
| | No | 441 | 33.1 | 260 | 100 |
| | Yes | 891 | 66.9 | 0 | 0.0 |
| Socio-economics | Education | | | | |
| | Elementary only | 121 | 9.1 | 35 | 13.5 |
| | Intermediary | 660 | 49.6 | 122 | 46.9 |
| | Graduate | 551 | 41.4 | 103 | 39.6 |
| | Economic activity | | | | |
| | Paid work | 444 | 33.4 | 107 | 41.2 |
| | Looking after home | 134 | 10.1 | 23 | 8.9 |
| | Unemployed | 22 | 1.7 | 3 | 1.2 |
| | Retired | 711 | 53.4 | 123 | 47.3 |
| | Other | 20 | 1.5 | 4 | 1.5 |
| | Financial constraints | | | | |
| | Constrained | 253 | 19.2 | 59 | 23.7 |
| | Intermediary | 668 | 50.6 | 114 | 45.8 |
| | Relatively unconstrained | 400 | 30.3 | 76 | 30.5 |
| Social support | Single | | | | |
| | No | 790 | 59.3 | 159 | 61.2 |
| | Yes | 542 | 40.7 | 101 | 38.9 |
| Smoking | Smoking status | | | | |
| | Never smoker | 903 | 68.0 | 157 | 60.4 |
| | Ex-smoker | 222 | 16.7 | 38 | 14.6 |
| | Current smoker | 204 | 15.4 | 65 | 25.0 |
| Alcohol | Alcohol use disorder | | | | |
| | Non-drinker (past year) | 136 | 10.2 | 25 | 9.6 |
| | Low (Audit score <8) | 1,163 | 87.6 | 224 | 86.2 |
| | High (Audit score 8+) | 29 | 2.2 | 11 | 4.2 |

| | | | | | |
|---|---|---|---|---|---|
| | Alcohol intake | | | | |
| | Non-drinker | 136 | 10.2 | 25 | 9.6 |
| | <2.5g/day | 788 | 59.3 | 149 | 57.3 |
| | 2.5-6.9g/day | 292 | 22.0 | 56 | 21.5 |
| | 7-14.9g/day | 73 | 5.5 | 18 | 6.9 |
| | 15-20.9g/day | 22 | 1.7 | 5 | 1.9 |
| | 21+g/day | 18 | 1.4 | 7 | 2.7 |
| | | | | | |
| Exercise | Physical activity | | | | |
| | Inactive | 102 | 7.8 | 17 | 6.7 |
| | Moderately inactive | 125 | 9.5 | 30 | 11.8 |
| | Moderately active | 771 | 58.7 | 142 | 55.7 |
| | Active | 316 | 24.1 | 66 | 25.9 |
| | | | | | |
| Health | BMI class | | | | |
| | Normal/under weight | 436 | 32.8 | 94 | 36.3 |
| | Overweight | 440 | 33.1 | 73 | 28.2 |
| | Obese | 455 | 34.2 | 92 | 35.5 |
| | | | | | |
| | Self-rated general health | | | | |
| | Poor/fair/good | 792 | 59.6 | 126 | 48.5 |
| | Very good/excellent | 537 | 40.4 | 134 | 51.5 |
| | | | | | |
| | Chest pain | | | | |
| | No chest pain | 704 | 54.4 | 159 | 64.1 |
| | Non-anginal chest pain | 374 | 28.9 | 54 | 21.8 |
| | Grade 1 angina | 175 | 13.5 | 33 | 13.3 |
| | Grade 2 angina | 42 | 3.2 | 2 | 0.8 |
| | | | | | |
| | CVD risk 10yr SCORE | | | | |
| | <1% | 653 | 49.9 | 144 | 56.5 |
| | 1-4.9% | 535 | 40.8 | 77 | 30.2 |
| | 5+% | 122 | 9.3 | 34 | 13.3 |
| | | | | | |
| | Hypertension class | | | | |
| | Normotensive | 628 | 47.6 | 140 | 54.9 |
| | Controlled hypertension | 304 | 23.0 | 24 | 9.4 |
| | Uncontrolled hypertension | 219 | 16.6 | 38 | 14.9 |
| | Untreated hypertension | 169 | 12.8 | 53 | 20.8 |
| | | | | | |
| | Hypertension aware | | | | |
| | No | 708 | 53.2 | 175 | 67.3 |
| | Yes | 624 | 46.9 | 85 | 32.7 |
| | | | | | |
| | Diabetic | | | | |
| | No | 1,198 | 89.9 | 242 | 93.1 |
| | Yes | 134 | 10.1 | 18 | 6.9 |

| | | | | | |
|---|---|---|---|---|---|
| | CKD | | | | |
| | No | 1,280 | 96.1 | 245 | 94.2 |
| | Yes | 52 | 3.9 | 15 | 5.8 |
| | Any care-intensive condition[a] | | | | |
| | No | 593 | 44.5 | 155 | 59.6 |
| | Yes | 739 | 55.5 | 105 | 40.4 |
| Medication | Polypharmacy 5+rx | | | | |
| | No | 1,248 | 93.7 | 258 | 99.2 |
| | Yes | 84 | 6.3 | 2 | 0.8 |
| | Non-opioid analgesics | | | | |
| | No | 1,315 | 98.7 | 256 | 98.5 |
| | Yes | 17 | 1.3 | 4 | 1.5 |

[a]) Care-intensive conditions included: CKD, chronic bronchitis, cancer, asthma, rheumatoid arthritis, osteoarthritis, migraine, opioid analgesics use.

**Table S5** Multivariable logistic regression analysis of a) no recent primary care contact (primary care visit in past year or recent general health check attendance) versus those with contact in CVD-free 40-69 year olds; b) no primary care visit in past year versus those with primary care visit in past year in CVD-free 40-69 year olds: age-adjusted odds ratios (AOR) by 10-year CVD risk class (SCORE).

| Group | Outcome | 10-year CVD Risk | Age-adjusted OR (Main analysis) | P-value | Age-adjusted OR* (no primary care visit/contact, but hospital visits) | P-value |
|---|---|---|---|---|---|---|
| Male | a) No primary care visit in past year nor recent general health check attendance | <1% | Ref | | Ref | |
| | | 1-4.9% | 1.03 (0.67;1.59) | .890 | 1.04 (0.67;1.61) | .872 |
| | | 5+% | **2.08** (1.15;3.76) | .016 | **2.21** (1.21;4.02) | .010 |
| | b) No primary care visit in past year | <1% | Ref | | Ref | |
| | | 1-4.9% | 1.10 (0.72;1.66) | .662 | 1.10 (0.73;1.67) | .649 |
| | | 5+% | **2.03** (1.15;3.59) | .014 | **2.12** (1.20;3.76) | .010 |
| Female | a) No primary care visit in past year nor recent general health check attendance | <1% | Ref | | Ref | |
| | | 1-4.9% | 1.05 (0.65;1.69) | .852 | 1.00 (0.61;1.63) | .995 |
| | | 5+% | **2.58** (1.27;5.23) | .009 | **2.52** (1.22;5.17) | .012 |
| | b) No primary care visit in past year | <1% | Ref | | Ref | |
| | | 1-4.9% | 1.21 (0.82;1.79) | .344 | 1.18 (0.79;1.76) | .414 |
| | | 5+% | **2.53** (1.40;4.58) | .002 | **2.33** (1.27;4.27) | .006 |

*) Sensitivity analysis taking into account that 5.5% of those who did not see a primary care doctor but did visit a hospital in the past year.
